# WNT signaling mechanisms in nociception and sensitization of afferent neurons

**DOI:** 10.1186/2193-1801-4-S1-L48

**Published:** 2015-06-12

**Authors:** Manuela Simonetti

**Affiliations:** Heidelberg University, Germany

**Keywords:** Wnt signalling, DRG, nociception

Wnt signalling represents an ancient and highly versatile signalling system, which plays diverse critical roles in embryonic development. Sensory neurons of the dorsal root ganglia require Wnt signalling for initial cell fate determination as well as patterning and synapse formation over later development. Recent studies have functionally linked mis-regulation of Wnt signalling to cancer, bone disorders and abnormal synaptic function in adult life. We will discuss a novel, functional role of Wnt signalling pathways in sensitization of peripheral adult sensory neurons in a pathophysiological context, and the underlying molecular mechanisms. Using an interdisciplinary approach, through different technique spanning molecular, genetic, and behavioural experiment in vitro as well as in vivo pain models in mice, we show that Wnt3a is able to recruit different Wnt-pathways, to alter pain sensitivity in a modality-specific manner, acting via intracellular kinases in peripheral nerves. We found evidence for an intriguing dichotomy of non-canonical signalling pathways in mediating mechanical and thermal hypersensitivity. Indeed, while the calcium pathway is mainly involved in thermal hypersensitivity, mechanical hypersensitivity is driven by the planar cell polarity pathway. Interestingly we did not find clear functional evidence for the canonical Wnt signalling pathway in Wnt3a-induced sensitization of nociceptors. Finally, we will provides evidence for a translational potential for targeting peripheral Wnt signalling in tumour-nerve interactions and pathological pain hypersensitivity, paving the way for therapeutic interventions. Acknowledgements This work was supported by grants from the Association of International Cancer Research and the Deutsches Forschungsgemeinschaft to R.K. R.K. is a principal investigator in the Excellence Cluster CellNetworks of Heidelberg University and M.S. was supported by the CellNetworks Postdoctoral Fellowship program.

